# What the Profession Owes Dr. Taggart

**Published:** 1911-11-15

**Authors:** L. S. Tenney

**Affiliations:** Chicago


### WHAT THE PROFESSION OWES DR. TAGGART.

BY L. S. TENNEY, D.D.S., CHICAGO.

I wish it were possible to accurately estimate the monetary value of the casting process to our profession. It is a very difficult thing to do, but I wish to call your attention to my own experience, to make an estimate from that, and leave you to judge for yourselves whether or not you consider me far out of the way in that estimate. You all know that after a man has practiced dentistry for some eight or ten years in a given community, his practice ordinarily becomes settled and stable: In my own case this was true to a remarkable degree, and after I had practiced for the length of time stated my practice did not vary from year to year more than a few hundred dollars, and had settled down to such a degree that I came to depend upon a certain income almost as surely as though I had been a commercial man with a fixed salary. In 1907 I noticed a marked increase in the proceeds of my practice. For a time I thought but little about it but after it had continued for a year I began to look around for its cause, and then I discovered that it was due entirely to the new method of operating. It was due to this for two reasons: First, because I could command larger fees for my cast than I could for my foil fillings, and second and more important because I found that I was inserting a very large number of inlays in positions which I had formerly used cheaper fillings, namely in frail and badly broken down teeth, and in cavities extremely difficult of access.

Now I am aware that in country practices, the increase from the casting method would probably not be as great as it has been in my case. On the other hand many men have informed me that their practice had increased still more than mine, but to keep well within the bounds of a reasonable estimate I find that if I take one-tenth of the amount my practice has increased and multiply it by the number of dentists in this country namely forty thousand, I arrive at an estimate of twelve millions of dollars annually which our profession is profiting as a result of the introduction of the Taggart inlay.

I wish to ask you now what have we given him in return for this rich harvest we are gathering? What has been our attitude toward this man who has thus enriched us? I do not hesitate to say that it has been one of absolute coldness and indifference, and that from the day of the introduction of this method up to the present moment we have, as a profession, ignored every just claim.

To deal thus with a man in the business world might possibly be expected. There men neither give nor expect to receive very much consideration. Fierce competition pervades it, and the vast business institutions that here surround us represent the survival of the strongest, but in dentistry we hold up a code of ethics as an evidence of the superiority of our methods over those of the man of business. When a business man invents or comes into the possession of some idea, some process, some means, by which he can gain advantage over his competitors, he seizes upon it and utilizes it and the world commends him for his genius, but when a dentist as a result of years of sacrifice and toil invents a process of incalculable benefit to us all and then asks us "What am I to receive as my compensation," we flaunt in his face a beautifully written document and we say, "Behold our code: Think of what you have done for humanity This must be your compensation."

Gentlemen, during the twenty years that I have been in the practice of dentistry no man can say that I have ever wandered one inch from the straight path of professional ethics, but when I look about me and see such injustice as this, I sometimes pause and inquire: "What is this code that enables me to seize and appropriate the product of a man's toil and the product of a man's genius, and return him nothing but a few honeyed words of commendation!" This is not ethics. It is not justice. It is confiscation. If ethics means anything to me it means that we should treat each other with honesty, with fairness, and with consideration nor can I understand that rule of conduct that says I may receive all and give nothing.

It has been said and it is probably true that had Dr. Taggart been a little more tactful, had he, before describing his process, have surrounded it with proper safeguards, had he seen to it that there were a large number of machines ready for the market he might have profited from the sale of them. Do men who make these statements recall that Dr. Taggart has always been a man of high professionalism; that from the earliest days of his practice he has been a constant attendant upon local, state and national meetings; that at those times he has contributed richly with papers, discussions and clinics? Do they recall that throughout his whole career he has taken an active part in whatever pertains to professional progress? Such is, in fact, the case, and I for one am generous enough to believe, his whole career justifies me in the belief, that the reason Dr. Taggart did not take the precautions men say he should have taken, was because he was imbued with that spirit of professionalism which blinded him to its necessity. He did not surround himself with means of protection because in his eagerness to do something for that profession for which he had already done so much, he overlooked it. He did not suppose that he had to protect himself from his friends. He belonged to a profession, and professional men have a code of ethics. Yes, men tell us that Dr. Taggart might have profited by his inventions had he been a little more tactful. Is this not a plain admission that our code of ethics is to be observed only when a man has us by the throat?

When this process first came out it was admitted by practically everyone that the profession owed Dr. Taggart something, and then what a farce did we see enacted? From every section of the country we heard the cry, "Why yes we admit that we owe Dr. Taggart something, just tell us how much it is and we will pay it. We recognize our indebtedness to this man. All we want is to have the amount decided upon." Men begged our societies to come together and formulate some definite plan of action, but when at last there came the opportunity for which they had long sought, when Crouse and his committee went to Taggart with a proposition, a proposition so small that it seems to me they would have turned from his door, ashamed to present it; when Crouse and his committee went to Taggart with a proposition and Taggart accepted it; and then when Crouse came to the profession and said "This troublesome matter is at last settled. All you have to do is to send twenty-five dollars, the magnificent sum of fifteen dollars to go to Dr. Taggart, for which you will have the right to use this process for seventeen years and probably all the rest of your life; and ten dollars to pay your membership fee in the Dental Protective Association, to encourage, strengthen and help to maintain an organization that has protected you for twenty-five years and stands ready to protect you all the rest of your life if you will give it your support." When Crouse came to the members of the profession with that proposition did you see them rise as one man to accept it? Did you see any wild stampede to see who could get to the treasury first? Turn to the books and there you will find your answer. Turn to the books and there you will find that out of the forty thousand dentists of this country whose hearts were bursting with gratitude and who were begging for an opportunity to reward their benefactor, four hundred have accepted and the other thirty-nine thousand six hundred still have their purses wrapped up in a code of ethics, "for humanity's sake."

Gentlemen, if some individual who had no interest in this profession except a mercenary one, were to attempt to impose upon and exploit us, we would rise as one man and fight him to the bitter end, but to deal in this manner with a man who is one of us, who has always been one of us, and who has devoted some of the best efforts of his life to our interests, to deal in this manner with such a man seems like an outrage upon every sense of justice, and if at some future time some other dentist shall invent some other process of equal value to us, and if, looking over the history of this case, he shall decide that the only course for him to pursue is to utterly disregard ethics and surround his process with every safeguard known to the law, who is there in this room or in this profession who could criticise his action? What is to be the solution of this problem? I have none to offer. Men more competent than I have attempted to solve it and failed, but since Dr. Taggart, as dentists have told us, was so indiscreet as to read a paper before a dental society without taking precautions to protect himself against his own professional brethren; since he was so unwise, as dentists have told us, as to read a paper before a representative body of professional men without first making sure that the walls of some great factory were creaking and bursting under the combined weight of a thousand machines, in order that the moment he had finished, he might rush there quickly, hurriedly, before we could confiscate his ideas; since all this is true I see no solution of the problem except through recourse to the law, for as I said at the beginning, "No question is ever settled until it is settled right."—*Review.*

